# Dry eye disease seasonal pattern and climatic association in Saudi Arabia using Google Trends

**DOI:** 10.1371/journal.pone.0339170

**Published:** 2025-12-19

**Authors:** Abdulaziz S. AlHarthi

**Affiliations:** Ophthalmology Division, Department of Surgical Specialities, College of Medicine, Majmaah University, Majmaah, Saudi Arabia; Canadian University Dubai, UNITED ARAB EMIRATES

## Abstract

**Purpose:**

The study aims to investigate the dry eye search term pattern in Saudi Arabia using Google Trends, and to explore the association of weather changes on dry eye disease (DED) search interests.

**Methods:**

Time series analysis for data that were collected from Google Trends (GTs) on period from January 2011 to October 2024 using Arabic term for Dry Eye with setting allocated in Saudi Arabia. Seasonality was evaluated using Fourier terms in ARIMA regression model. Monthly variation was further evaluated. Climate factors, mean surface air temperature, relative humidity and accumulated precipitate were incorporated into ARIMAX model to find environmental relationship with DED. Kruskal-Wallis test was performed to confirm significant finding in monthly variation.

**Results:**

Dry eye disease (DED) related search term demonstrates significant monthly differences (*p* = 0.008). Monthly effect ARIMA model (R² = 0.93) identified sustained high season from February through August higher than January. June represents maximum annual peak. A significant upward trend of 0.48 per month in RSV is also noted over the 13-year period (*p* < 0.001). In ARIMAX model, relative humidity is most important associated factor with dry eye search activity (β = −0.26, *p* = 0.002).

**Conclusion:**

This study is the first evidence of seasonality of DED using Google Trends (GTs) in Saudi Arabia and highlighting the growing public health concern of DED. Understanding the disease pattern can aid public health implications to decrease risk of DED. This finding can serve as valuable reference to supplement traditional methods.

## Introduction

Dry Eye Disease (DED) is a common eye disorder worldwide, with characteristics ocular symptoms that range from mild ocular surface discomfort to more symptomatic that may have a substantial impact on individuals’ quality of life and vision [1]. DED is a multifactorial disease cause alteration of tear film homeostasis and broadly classified according to its pathophysiology into aqueous deficient dry eye (ADDE) and evaporative dry eye (EDE) [2]. DED is chronic ocular disease affecting hundreds of millions of people worldwide with global prevalence reported rates varying widely from 5% to 50% [3]. Moreover, In the Arab countries, the estimated prevalence of DED is relatively in high ranging percentage (10–73.4) in compared to other parts of the world [4,5]. The increase of prevalence of DED was suggested to be linked with the high prevalence of rheumatoid arthritis, diabetes mellitus, and the heavy use of age-related systemic and topical medications in elderly people along with environmental factors [4,5]. In addition, the prevalence of DED can vary widely from one population to another. The differences are attributable to different definition, type of diagnostic test, individual age, lifestyle, and geographical location across studies [4].

The ocular surface is in direct contact with surrounding air; consequently, the environmental factors can play a role in DED by accelerating the tear film evaporation rate. Comprehensive report by Tear Film & Ocular Surface Society (TFOS) Lifestyle workshop showed that environmental conditions can interact with ocular surface and increase risk of DED; such factors include low humidity, high/low temperature, Higher wind speed, high attitude, ultraviolet light exposure, and exposure to air pollution [6]. Tear Film & Ocular Surface Society reported in Dry Eye Workshop II have also called for further research to elucidate more on the influence of climate and environmental factors on DED [2].

Understanding the burden of DED prevalence and pattern is not exactly estimated worldwide. The use of Google Trends data might reflect the specific season pattern better than hospital-based data since patients with undiagnosed DED might be difficult for them to visit the hospital, and most hospitals is not easily accessible because it requires referral letter and prior reservation. Google Trends (GTs) (http://google.com/trends/) is an online open source that provides estimation of users’ interest behaviour by analysing relative search volume for terms over time periods. Google Trends (GTs) is a powerful tool that has been used to observe public interest to obtain information throughout the internet, which can be used as surveillance tool to predict prevalence, assess seasonal variation or infectious disease tracking [7,8]. The Relative search volume (RSVs) is normalized and presented on scale from 0 to 100 based on popularity of all searches on all topics and duplicate searched from the same user is not included [9]. This science is known as infodemiology which use digital data to inform the public health [7]. For DED, these data analysis can show a more real time population interest and potential pattern of DED symptoms.

In this study we explore time-series analysis on the public internet searches of dry eye to better understand the likely evidence of seasonal variation on the prevalence of dry eye disease (DED) and find association with weather changes. Such findings can help in eye care recommendation and provide reference for conducting epidemiologic research on DED. To our knowledge, this study is the first to describe seasonality of dry eye in Saudi Arabia using Google Trends tool.

## Materials and methods

### Overview

Seasonality pattern is regular and predictable data changes which peak or trough at every certain period of time every year (month/season as in this case). While the trend is the increase or decrease in data over historical time. Meteorological season was defined as summer start from June to August, fall start from September to November, winter start from December to February, and spring start from March to May.

### Google trends and environmental factors data

We extracted monthly aggregated normalized relative search volume data (RSV) (scale 0–100) from Google Trends as CSV file in September 2024 for the search term in Arabic language “جفاف العين” for dry eye. The location is set in Saudi Arabia for the period from January 2011 to October 2024, for web searches and without category selection. The selected Arabic translation term is widely recognized for dry eye. Google Trends does categorize and aggregate related searches, variations in spelling and related terms into determined topic. Therefore, the data we used for the selected term likely to capture wide range of queries under the topic of dry eye. We collected data for Alternative and top related Queries to verify adequate representation to dry eye term.

For historical environmental factors data, we collected monthly aggregated average mean surface air temperature (°C), monthly relative humidity (100), and monthly Aggregated accumulated precipitation (100) at national level for Saudi Arabia spanning from January 2011 to December 2023. Data were retrieved from ERA5 0.25-degree dataset in World Bank Group website (https://climateknowledgeportal.worldbank.org/).

### Statistical analysis

The monthly relative search volume was formatted as univariate time series. Data series were decomposed into trend, season, and errors factors, which represent time changes, periodic changes and random changes, respectively. To validate the presence of monthly/seasonal variation, the RSV was initially examined by non-parametric Kruskal-Wallis test.

Prior to model fitting, Stationarity test was performed using Augmented Dickey-Fuller (ADF) and seasonal stability using Canova-Hansen. For model selection, we used auto.arima function to find the minimum corrected Akaike information criterion (AICc). The selected model was based on parsimonious model principle for simple and less prone to overfitting noise and residual diagnostic (Ljung-Box *p >* 0.05).

To quantify the seasonality, Fourier terms in Autoregressive Integrated Moving Average (ARIMA) regression modelled. Monthly variation with month of January as reference baseline was further investigated. The model was evaluated by assessing the residual normality, autocorrelation function (ACF) plot, and Ljung-Box test. To validate the selected search term for analysis, Spearman correlation test was performed between dry eye term and top-related Arabic queries. To learn significant association between relative search volume (RSV) for dry eye and highly correlated weather factors, ARIMAX regression model incorporating average mean air temperature, relative humidity, and precipitate as exogenous regressors. Multiple model analyses were used to find significant factors that were associated with dry search intent volume.

To estimate the impact COVID-19 restriction’s, binary variables coded (0/1) for the intervention period from March 2020 to March 2022 was then incorporated in the monthly fixed effect model.

The R software, version 4.4.1 (R Development Core Team) was used to make the analysis on the GTs collected data with forecast package using auto.arima() function and to further assess seasonality using seastest package. Across all analysis, the significant p value was set < 0.05.

### Ethical consideration

Google trends data is open public-source. Climatic data from the World Bank Group is under Creative Commons Attribution 4.0 International (CC BY 4.0) Therefore, No institutional Review Board (IRB) approval was needed.

## Results

**Fig 1:**
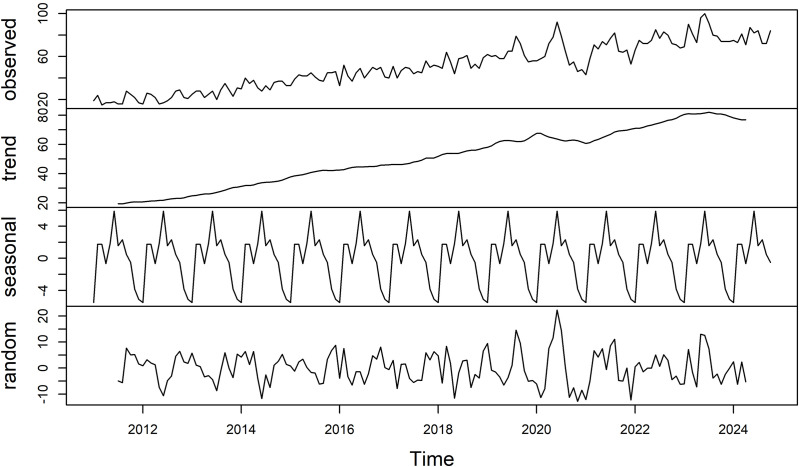
Decomposition timeseries data of dry eye search term in Saudi Arabia from 2011 to October 2024. The data series of monthly relative search volume (RSV) obtained from Google trends platform for the Arabic search term for dry eye. We can see the three decomposed components in the bottom three panels.

(Fig 1) shows the finding of Google Trends data decomposition for the Arabic term, which suggests the occurrence of dry eye disease (DED) have obvious seasonal components, and the trend of search is increased throughout the study period and height of cycles appears to be increasing. To explore the presence of seasonal components non-parametric tests were conducted. The Kruskal-Wallis test (H(11, N = 156)= [25.47], *p* = 0.008), which confirm that relative search volume for dry eye term in Arabic is significantly different across the months of the year.

### ARIMA model construction

**Fig 2:**
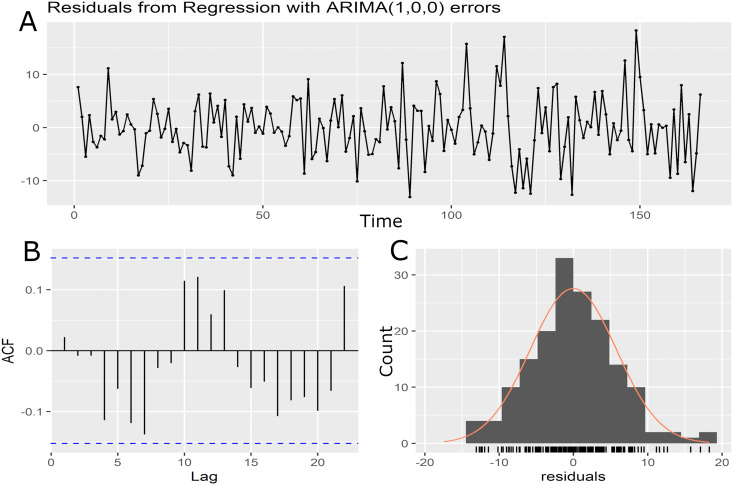
The residual diagnostic plot of Month effect ARIMA. **(A)** Residuals of relative search volume for dry eye using ARIMA Model (1,0,0) reveal white noise, **(B)** Autocorrelation function (ACF) of the residuals with lack of significant correlation, **(C)** Histogram of residuals series suggests the distribution is approximately normally, supporting the ARIMA model adequacy.

To identify the seasonal variation, an ARIMA model was used to determine the pattern of months variations. Initially, Augmented Dickey-Fuller (ADF) test result with trend was (−6.737, *p* = 0.01) indicating the data in this study is trend-stationary. Canova-Hansen (1.7424, *p* = 0.4216) is overall stable, Indicating data series exhibit type of deterministic seasonality rather than stochastic. By using auto.arima() function, The determined month effect ARIMA (1,0,0) model established good fit to the data. The model passed the box Ljung test (*X2* = 11.392, *p* = 0.2498), Durbin-Watson (DW = 1.94, *p* = 0.343), and the residual was normally distributed and no spikes on autocorrelation, which indicates that the fitting residual is approximately white noise and there is no heteroskedasticity (Fig 2). The accuracy of ARIMA/ ARIMAX model fit was satisfactory.

### Seasonal and monthly variation effect

The summary results of the ARIMA model are presented in Table 1. The analysis reveals annual seasonality exhibit sustained high season from February through August; all months were statistically significant above the reference month (January) except for April. The annual maximum peak is in June with amplitude of 3.52 RSV unit. February showed strong elevation onset from January baseline. Furthermore, There is 0.48 RSV unit of relative volume increase in search intents over time during the study period from 2011 to 2024 (β = 0.475, 95%CI [0.33, 0.62], *p* < 0.001), that mean the RSV increases by 0.48 RSV units per month. Regarding impact of COVID-19 quarantine in the relative search volume for dry eye, there was no significant effect (β = 0.47, 95%CI [−4.57, 3.64], *p* = 0.822).

**Table 1:** The result of ARIMA (1,0,0) model of the different month of the year for dry eye search term in Saudi Arabia.

Variable	Coefficient (β)	Std. Error	t-Statistic	95% CI	P-value
**Monthly Effects (vs January)**					
**Intercept**	10.364	3.284	3.156	[3.874, 16.854]	< 0.001*
**February**	7.081	1.871	3.785	[3.384, 10.777]	< 0.001*
**March**	6.308	2.383	2.647	[1.599, 11.017]	0.009*
**April**	4.109	2.713	1.515	[-1.251, 9.469]	0.132
**May**	6.911	2.504	2.76	[1.963, 11.860]	0.007*
**June**	10.002	2.65	3.774	[4.765, 15.239]	< 0.001*
**July**	6.595	3.003	2.196	[0.660, 12.530]	0.030*
**August**	6.336	2.699	2.347	[1.002, 11.669]	0.020*
**September**	4.586	2.521	1.819	[-0.396, 9.568]	0.071
**October**	4.498	2.829	1.59	[-1.092, 10.088]	0.114
**November**	1.187	2.666	0.445	[-4.080, 6.455]	0.657
**December**	0.07	2.109	0.033	[-4.098, 4.239]	0.973
**Temporal Effects**					
**Time**	0.475	0.071	6.65	[0.334, 0.616]	< 0.001*
**Time²**	−0.0004	0.0004	−0.954	[-0.001, 0.001]	0.342
**AR(1)**	0.471	0.073	6.491	[0.327, 0.614]	< 0.001*

Note: The coefficient for the intercept is the benchmark for January. months are relative to January for comparisons. AR(1) is first order autocorrelation. * indicate *p* < 0.05, Model fit and diagnostic: R² = 0.925, AIC = 1013.14. Observational number = 166.

### Top related search term for dry eye in Saudi Arabia and its correlation with main study search term

**Fig 3:**
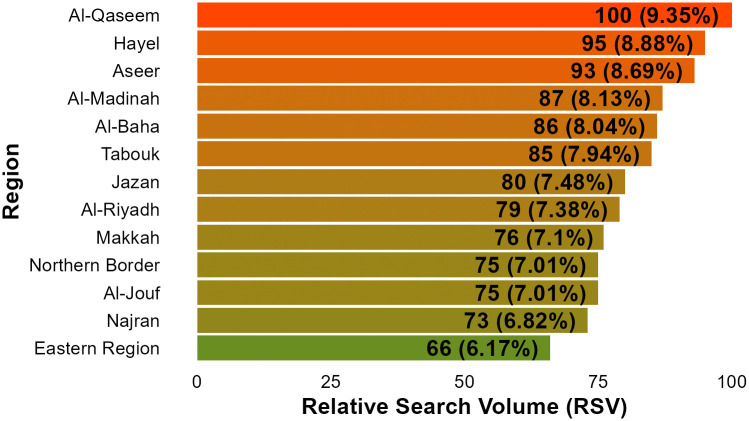
Dry eye search term popularity by region in Saudi Arabia with percentage distribution. We can see the data indicate Al-Qaseem region has a higher probability for dry eye searches. While Eastern region search popularity represent 34% lower searches for dry eye in compared to Al-Qaseem region.

Analysis of top related search term over the study period Table 2, revealed searches interests centres around symptoms, causes, complications and management of dry eye. The consistent positive strong correlation confirms that the similar search interest behaviour pattern across related queries. Additionally, the strong positive correlation between dry eye search term and dry eye unified topic (Spearman ρ = 0.51, *p* < 0.001), support the strength of using the representative search term for analysis. Regional variation in search popularity was also observed (Fig 3).

**Table 2:** Top related queries from GTs for dry eye in Arabic term in Saudi Arabia.

Category	Top related search term	Data	Total top related search interest percentage (%)	Correlation
**Symptoms**	Eye symptoms	100	17.51	0.063
	Dry eye symptoms	98	17.16	0.882***
	eye pain	15	2.63	0.944***
	Eye headache	13	2.28	0.938***
	Sign of dry eye	11	1.93	—
	Symptoms of dry eye	10	1.75	—
	dry eye and headache	6	1.05	—
		**253**	**44.31**	
**Causes**	causes of dry eye	72	12.61	0.760***
	cause of dry eye	36	6.30	0.646***
	what is cause of dry eye	7	1.23	—
		**115**	**20.14**	
**Management**	dry eye drop	33	5.78	0.590***
	eye drop	33	5.78	0.780***
	dry eye drops	18	3.15	—
	dry eye treatment at home	6	1.05	—
		**90**	**15.76**	
**Complications/ inflammation**	Eye inflammation	14	2.45	0.906***
	Complications of dry eye	8	1.40	—
		**22**	**3.85**	
**Other Anatomical dryness**	body dryness	8	1.40	0.890***
	dry mouth	7	1.23	0.912***
		**15**	**2.63**	
**General searches**	Sever dry eye	15	2.63	—
	Dry eyes (plural)	14	2.45	—
	Dryness around the eye	14	2.45	—
	Dryness under the eye	13	2.28	—
	red eye	7	1.23	0.942***
	what is dry eye	7	1.23	—
	eye allergy	6	1.05	0.823***
		**76**	**13.31**	

Top related search term associated with dry eye in Saudi Arabia were categorized. Data are presented as normalized relative search volume (RSV) from 0 to 100 for the most popular query for period from January 2011to October 2014**.** While the percentage represents each related term proportion of cumulative search related activity. Note: Spearman’s rho (ρ) * *p* < .05, ** *p* < .01, *** *p* < .001. Dash (-) indicates correlation is not calculated as relative search volume (RSV) is low search frequency or relative interest equal to zero.

### Climate factors association

**Fig 4:**
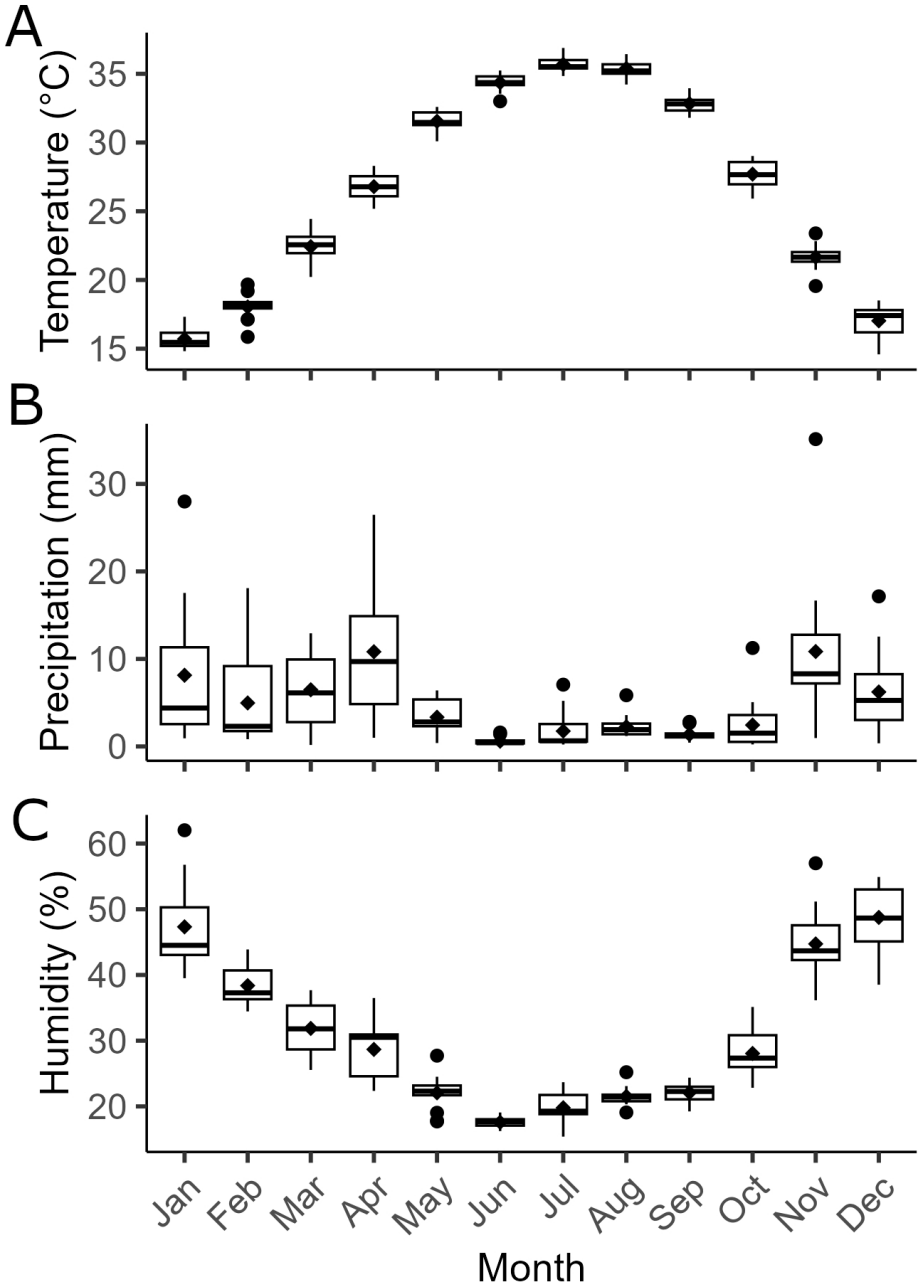
Box and Whisker chart of Saudi Arabia climate characteristics. **(A)** Average Mean Surface Air Temperature, **(B)** Precipitation, **(C)** Relative Humidity. These historical monthly climate data collected for period from January 2011 to December 2023.

The monthly average of meteorological factors was shown in (Fig 4). Temperature, humidity and Aggregated accumulated precipitation reveal characteristic of hot and dry weather represent typical of arid or semi-arid climate with means of 26.6°C, 30.9% and 4.94 mm, respectively. Humidity and precipitate were significantly related to relative search volume for dry eye in separate analysis Table 3. Precipitation was associated with decreased search intent for DED of 1.00 RSV unit per 5 mm of precipitation. 10% of increased in Relative humidity were associated with 2.6 reduction of search intent of DED.

**Table 3:** ARIMAX regression model analysis between dry eye search term and meteorological factors.

Variable	Model 1		Model 2		Model 3		Combined Model	
	β (95% CI)	p-Value	β (95% CI)	p-Value	β (95% CI)	p-Value	β (95% CI)	p-Value
**Relative Humidity**	−0.256 [−0.419, −0.093]	<0.002***	—	—	—	—	−0.293 [−0.536, −0.050]	0.018*
**Precipitation**	—	—	−0.213 [−0.369, −0.061]	0.006**	—	—	−0.087 [−0.292, 0.118]	0.404
**Temperature**	—	—	—	—	0.349 [−0.214, 0.9133]	0.224	−0.159 [−0.553, 0.207]	0.395
**Model fit and diagnostic**								
**R²**	0.925		924		0.921		0.919	
**Adjusted R²**	0.921		0.920		0.917		0.915	
**AIC**	999.5		1001.28		1007.05		1008.08	

Note: *** *p* < 0.001, ** *p* < 0.01, * *p* < 0.05. Dashes indicate variable excluded from model. The best fit model for separate ARIMAX models was (2.0.3), while for combined model was ARIMAX (1.0.0). All models include trend time. Total monthly observation = 156. box Ljung test: *p* > 0.05 for all models. AIC, Akaike Information Criterion.

## Discussion

The widespread use of smartphones and availability of internet become part of daily life activity. This made intent search data can reflect on particularly public health interest. In Saudi Arabia, Google is one of the top used search engine to seek for online informations [10]. Google Trends (GTs) offers a powerful infodemiology tools; and data analysis can be used for disease surveillance and identify public interest pattern of disease to make healthcare care policy [7].

Previous studies have concluded the presence of seasonal pattern in DED. Study in US reported significant higher internet search volume in spring and being lowest in fall season [11]. However, in China the prevalence of dry eye was slightly higher in warm season than cold season [12]. On the other hand, Van Stten et al [13] have described the seasonality for dry eye complaints in Europe to be during winter and summer, with environmental characteristics such as hot and cold as well as wind to be the most common triggering factors. Other studies in China indicated seasonal peak in winter [14,15]. Other investigations, did not support the evidence for seasonality in DED except for seasonal variation in Schirmer test and tear meniscus height parameters [16]. Variability of the reported findings concerning the seasonal pattern in DED between the studies or not finding association with DED could be linked to different geographical location. It is not only the seasonal pattern, but also the regional differences in prevalence of DED. It was suggested that climate, weather parameters, environmental exposures, and socioeconomic considerations as potential causes. However, validating relationship between large number of potential confounders and DED prevalence is challenging [17].

Geographical variation in climate and environmental can be associated with variation in the prevalence of DED. Factors that play a role include temperature, humidity, rainfall, and air pollution. The Arab peninsula does experience nearly 15 heat waves lasting 2–5 days annually mainly during summer months [18]. The drought based on the rainfall threshold occur in dry season with month of June and September, respectively, are considered more prone to drought. In the beginning of wet season, the drought trend decreased number of drought, with the exception of February being higher than January and March, and month of April is the less prone to drought [19]. In regard to storms events, Autumn months have lowest number of sand and dust storms events due to stable winds condition, whereas 70% of storm events reported in spring and summer [20]. Rapid transition from wet to dry season (known as hydroclimate whiplash) might also be triggering factor for DED symptoms. These factors may hypothetically coincide with seasonality. Climate changes may contribute to the increased internet search volume of dry eye during summer months and February. As of our result, Relative humidity showed negative association with dry eye search interest. This indicates air moisturize may have led to decrease in relative volume of dry eye. Furthermore, our finding of steady increase of relative search volume over the study period could potentially linked to the ongoing climate change, temperature rise, or lifestyle changes. This may reflect public health concern for increased number of DED. It has been observed that in comparison of the current literature with previous studies from the Middle East showed increased prevalence of symptomatic dry eye disease [4]. Wearing sunglasses and avoiding going out during harsh weather should be emphasized in an effort to decrease the prevalence.

The variation in search ranking across different provinces in Saudi Arabia, further supports the role of geographical difference due to the role of environmental factors. The central regions of country (e.g., Al-Qaseem, Hayel), characterized by desert climate with hot temperature and low humidity, was in highest rank. In contrast, Eastern region of Saudi Arabia, characterized by humid coastal regions, was the lowest in ranking of search volume. This considerable variability across different locations in the country could be implicated by the influence of high humidity climate in Eastern region to reduce tear evaporation giving protective effect from DED. It has been shown that higher humidity has a strong correlation to improve corneal fluorescein staining and tear breakup time (TBUT) [21]. Prevalence of DED in Arab countries is not clearly established. In Saudi Arabia, The prevalence of DED demonstrated by studies using survey from different regions revealed variable prevalence rate ranged from 32.1% to 75.9% [22]. Interesting, the reported prevalence of dry eye in the Eastern province of Saudi Arabia (65.4%) was similar to the prevalence in Dubai [23]. Given potential similarity of geographical location. Due to divergence in climate and humidity across regions in Saudi Arabia, further research for the prevalence might elaborate more on the climate effects.

Additionally, behavioural, cultural and indoor environment may also contribute. In summer, individuals stay more time indoor, where indoor high temperatures, low humidity, and reliance on heavy use of air conditioners can adversely contribute to dry eye [24]. Individuals can perceive dry air by dry skin and dry eyes because of the direct contact with environmental air. Modifications of environment have also been demonstrated to decrease the alteration in tear film stability, for instance, it has been shown that elevation of low indoor humidity,within set points, is beneficial in decreasing the perception of dry air [25]. The air quality is fundamental for human life, and any pollution can have direct effect on health. Air pollutants such as PM2.5, PM10, NO2, CO and SO2, found to have significant association with increased DED outpatients visits [12,15]. Extended digital device exposure and other behavioural-cultural risk factors such as tobacco consumption, cosmetic use and contact lens are also found to be associated with higher prevalence of DED [26,27]. Emphasizing for more behavioural adaptation such as regular blinking and eyelid closure, especially during unavoidable risk of higher tear evaporation rate during the seasonal peak should be advised. During COVID-19 (coronavirus) pandemic, it has been demonstrated that DED increased due to potential longer digital screen exposure. However, in this study there was no significant shift in the trend of search volume in Google Trends during COVID-19 restriction period.

### Limitation

This study has limitations as the result is based on Google trends search volume data. First, the result is for the current period. Second, The reliability of Google Trends can be affected by media influence or public health campaigns with increased tendency to seek information online. Google Trends does not differentiate between distinct groups of patients, severity of dryness or if the symptoms were only transient feeling of dryness that does not meet the criteria for DED diagnosis. Moreover, Google Trends does not separate actual patients from individuals with general curiosity. Third, the data from Google Trends raise concern regarding selection bias toward internet users who have digital access and literacy for online search for health information. Potentially underrepresenting older populations, whereas the majority of dry eye disease were more common among them as reported in the literature. Fourth, our finding relied on single widely recognised Arabic term for dry eye, despite Google Trends aggregation and categorization of search query under unified topic, may not capture all colloquial spellings and symptoms related to dry eye disease.

## Conclusion

This study is the first study in Saudi Arabia that leverage GTs data to elucidate seasonal pattern of public interest in dry eye disease. Among climate factors, low humidity was the most important factor of increased dry eye search volume. Understanding the disease pattern can have timely public health awareness initiatives, public education on risk factors for DED and implementation of preventive strategies.

## Supporting information

S1 Data Dataset used for statistical analysis. (XLSX)
